# Management of Metastatic Renal Cell Carcinoma in a Tertiary Care Hospital

**DOI:** 10.7759/cureus.35623

**Published:** 2023-02-28

**Authors:** Sanjeev Kumar, Vishwajeet Singh, Mukul K Singh, Satya N Sankhwar

**Affiliations:** 1 Urology, King George’s Medical University, Lucknow, IND

**Keywords:** cytoreductive nephrectomy, overall survival, indian subcontinent, targeted therapy, international metastatic renal cell carcinoma database consortium, metastatic renal cell carcinoma

## Abstract

Background

The behavior of metastatic renal cell carcinoma (mRCC) is unpredictable and elusive. International Metastatic Renal Cell Carcinoma Database Consortium (IMDC) scores, histological subtypes, and targeted therapy predict survival and prognosis. However, there is a paucity of literature from the Indian subcontinent on mRCC outcomes. Therefore, this prospective study reports overall survival outcomes and complications due to targeted therapy of mRCC from a single tertiary care center.

Methodology

Between 2015 and 2020, 110 patients were included in the study. The treatment was based on the IMDC. Cytoreductive nephrectomy was done in 30 patients, and renal mass biopsy was done in 80 patients. Six were lost to follow-up after histopathological diagnosis, and targeted therapy was administered to 104 patients (sunitinib in 41, sorafenib in 33, and pazopanib in 30). During targeted therapy, six died within 30 days of treatment. The overall survival outcomes and complications due to targeted therapy were analyzed.

Results

The mean overall survival was 21.52 months with a 95% confidence interval of 17.04-25.98 months. Six variables significantly correlated with inferior survival in univariable Cox regression analysis. Weight loss, hemoglobin, platelet count, lung metastasis, and ≥2 visceral metastases were associated with poor outcomes. Performance status >2 and lung metastasis predicted poor outcomes in multivariate analysis. Overall survival was 24.52 months in clear cell carcinoma versus 21.39 months (13.32-29.45 months) in papillary cell carcinoma, which was not significant.

Conclusions

IMDC groups show significant differences in overall survival. The histological subtypes and types of targeted therapy did not differ in overall survival, and the presence of sarcomatoid differentiation correlated with poor prognosis concerning IMDC.

## Introduction

Renal malignancies are the third most frequent genitourinary malignancy, accounting for over 431,288 new cancer diagnoses and over 179,368 deaths worldwide each year [1-3]. Computed tomography (CT) and magnetic resonance imaging (MRI) have resulted in the increased diagnosis of early renal cell carcinoma (RCC) lesions in many individuals, and the five-year survival rate for early RCC detection is as high as 93%. Unfortunately, the five-year survival rate of RCC patients with metastases is only 12%. Because kidney tumors are so diverse, their behavior is unpredictable and elusive. Metastatic renal cell carcinoma (mRCC) exhibits several unusual features, including substantial hypervascularity, a high frequency of paraneoplastic syndrome, spontaneous regression of metastatic lesions following initial tumor resection, and late recurrence (>5 years). RCC shows a variety of metastasis patterns, with the lungs and bones remaining the most prevalent [3]. Although clear cell carcinoma is the most frequent histological subtype of RCC, accounting for 70-80% of cases, clinicians frequently rely on validated prognostic risk models for predicting survival, such as the Memorial Sloan-Kettering Cancer Center (MSKCC) risk score [4] developed during the cytokine era, and, later, the International Metastatic Renal Cell Carcinoma Database Consortium (IMDC) risk score [5] developed during the targeted therapy era. Patients with mRCC are divided into three risk groups, namely, favorable, intermediate, and poor [4,5]. Given its purpose, candidacy, and timing, cytoreductive nephrectomy (CN) is a moving target. It is possible that by including immune checkpoint inhibitors (ICIs) in the treatment repertoire, more patients will benefit from consolidative surgery CN, which has previously been reserved for patients with lower risk scores. The mRCC treatment landscape has changed throughout eras.

Despite scientific advancements that have resulted in increased progression-free survival (PFS) and overall survival (OS) rates, the majority of patients with mRCC will eventually develop progressive disease and succumb to cancer. Thus, research efforts are required indefinitely to expand information and expertise regarding mRCC to provide a holistic cure tailored to the needs of such individuals. In this study, we conducted a prospective analysis from a single center in northern India using targeted therapy of sunitinib, sorafenib, and pazopanib to investigate the survival rate and complication among the treated group, which may predict the treatment effect and investigate the treatment approach in the future for the north Indian population of mRCC patients.

## Materials and methods

Study design

The protocol of this prospective, observational study was approved by the Institutional Review Board of King George’s Medical University in Lucknow, Uttar Pradesh, India. All patients signed a written informed consent form. Patients who presented to the department during the study period (August 2015 to August 2020) were included. Those who refused to participate were excluded. The inclusion criteria included cases of metastatic renal tumors, whereas the exclusion criteria included patients with a primary focus of malignancy other than that of renal origin.

Data collection

Patient demographics, clinical presentation, radiological findings, hemograms, serum chemistries, clinical staging, and histopathology were documented. Individual treatment decisions were made in accordance with the specified guidelines. All included patients were monitored for three months. Clinical examinations, blood chemistries, and radiographic tests were performed at each follow-up visit. Adverse drug reactions were documented, and dose adjustments were made as needed.

Outcomes

OS was the primary endpoint. The duration from the beginning of the drug therapy to death or the last follow-up was designated as the survival time. The study lasted from January 2015 to December 2020. Complications or adverse medication responses were secondary outcomes.

Data analysis

Discrete variables were compared followed by the chi-square test. OS was estimated using the Kaplan-Meier test, and differences were determined using the log-rank test. A p-value of 0.05 was deemed statistically significant. Univariable and multivariable Cox regression analyses were performed to identify prognostic variables. All statistical analysis was performed using SPSS Software version 21.0 (IBM Corp., Armonk, NY USA).

## Results

**Figure 1:**
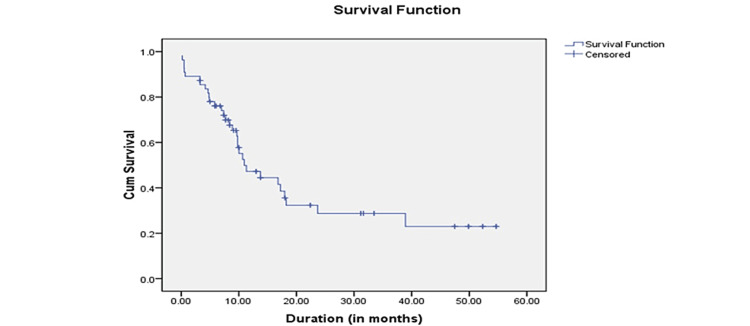
Kaplan-Meier curve for overall survival of patients.

This study included 110 mRCC patients. Table 1 summarizes the characteristics of patients with clinical tumor stage. The mean length of follow-up was 13.57 months. Patient care was categorized based on their IMDC score. Four patients from the favorable group and 26 patients from the intermediate group with good Eastern Cooperative Oncology Group (ECOG) scores (a total of 30 patients) received CN. Of the 30 patients, two were lost to follow-up after CN, and 28 mRCC patients underwent targeted therapy. In total, 80 mRCC patients with bad ECOG scores (n = 50 with poor IMDC scores, n = 30 with intermediate values) underwent renal mass biopsy (RMB). Four of the 80 patients were lost to follow-up after RMB, while the remaining 76 received targeted therapy. Six of the 76 patients died within 30 days of starting targeted therapy. The mean OS was 21.52 months, with a standard deviation of 2.282 months and a 95% confidence interval (CI) of 17.040-25.985 months. The median survival was 10.960 months, with a standard deviation of 1.861 months and a 95% CI of 7.312-14.608 months. The Kaplan-Meier curve for OS is shown in Figure 1.

**Table 1:** Characteristics of patients and tumors. ECOG = Eastern Cooperative Oncology Group; IMDC = International Metastatic Renal Cell Carcinoma Database Consortium

Characteristics of patients and tumor	N (%)
Median age (years)	50 years (21–72 years)
Male/Female	82 (74.5%)/28 (25.45%)
Symptomatic patients (flank pain or heaviness, lump, hematuria, weight loss, dyspnea, hemoptysis, bone pain, headache, seizures, jaundice)	110(100%)
Presence of comorbidities	28 (25%)
Laterality of tumor (right/left)	56 (51%)/54 (49%)
Venous thrombosis	36 (32.7%)
ECOG	
≤1	68 (61.8%)
>1	42 (38.2%)
Clinical T stage	
T1	3 (2.72%)
T2a	6 (5.5%)
T2b	20 (18.2%)
T3a	26 (23.6%)
T3b	20 (18.2%)
T3c	6 (5.5%)
T4	29 (26.3%)
Clear cell histology	94 (85%)
Sarcomatoid differentiation	16 (15%)
Papillary cell carcinoma	16 (15%)
Cytoreductive nephrectomy	30 (27.3%)
Preoperative angioembolisation	16 (14.54%)
IMDC score	
Favorable	4 (3.6%)
Intermediate	56 (50.9%)
Poor	50 (45.5%)
Number of metastatic sites	
1	68 (61.8%)
>1	42 (38.2%)
Metastatic sites	
Lung	86 (78.2%)
Bone	24 (21.8%)
Liver	24(21.8%)
Brain	4 (3.6%)
Retroperitoneal lymph nodes	54 (49%)
Targeted therapy	
Sunitinib	41 (39.42%)
Sorafenib	33 (31.73%)
Pazopanib	30 (28.85%)
Best response to therapy (RECIST v.1.1)	
Partial response	12 (12.2%)
Stable disease	16 (16.3%)
Progressive disease	70 (71.4%)

**Figure 2:**
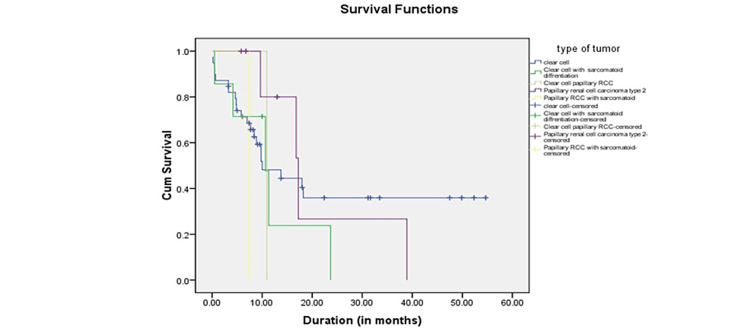
Kaplan-Meier curve for survival based on the histology type.

**Figure 3:**
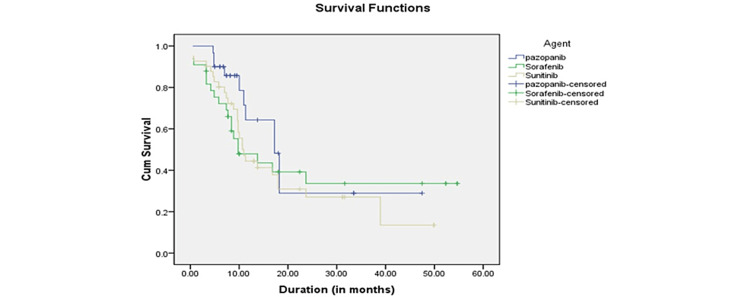
Kaplan-Meier curve for survival based on targeted therapy.

The OS in clear cell carcinoma was 24.52 months (18.74-30.31 months) compared to 21.39 months (13.323-29.457 months) in papillary cell carcinoma, which was statistically insignificant (p = 0.307). In both clear cell and papillary RCC, the presence of sarcomatoid differentiation reduced survival considerably. Figure 2 depicts the Kaplan-Meier curve for survival according to the type of histology. In addition, survival was analyzed based on the type of targeted therapy used. As shown in Figure 3, the mean survival time with pazopanib was 23.10 months (14.445-31.762), sorafenib was 23.96 months (15.481-32.432), and sunitinib was 19.205 months (13.638-24.773). The log-rank test was applied to the survival outcomes based on targeted therapy, and it was observed that the type of targeted therapy made no statistical difference in survival (p = 0.362).

**Figure 4:**
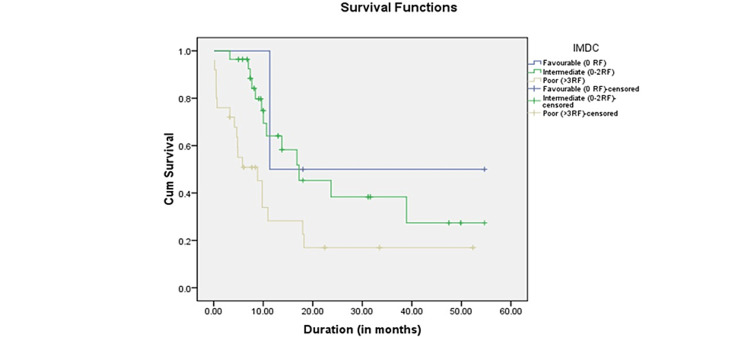
Kaplan-Meier curve for survival time based on the IMDC score. IMDC = International Metastatic Renal Cell Carcinoma Database Consortium

The survival study based on the IMDC score revealed that the favorable risk group had the highest survival and the poor risk group had the lowest. The favorable (-0 risk factor (RF)) group had a mean survival time of 33.000 months (11.763-54.237), the intermediate group (1-2 RF) had a mean survival time of 26.872 months (20.431-33.314), and the poor group (>3 RF) had a mean survival time of 14.399 (8.851-19.947) months. The p-value for comparing survival periods using the log-rank test was 0.001, which was statistically significant. Figure 4 depicts the Kaplan-Meier curve for survival time based on the IMDC score.

The univariable Cox proportional hazards regression analysis (UCPHRA) of the entire cohort revealed six independent factors or variables predicting poor survival (Table 2). These included ECOG Performance Status (PS) ≥2, hemoglobin <lower limit of normal (LLN), platelet count >upper limit of normal (ULN), lung metastasis, ≥2 visceral metastases, and weight loss. The multivariate Cox proportional hazard regression analysis (MCPHRA) revealed that ECOG PS ≥2 and lung metastases had statistically significant worse survival (Table 2).

**Table 2:** Univariable and multivariable Cox proportional hazards regression analyses. HR = hazard ratio; CI = confidence interval; ECOG PS = Eastern Cooperative Oncology Group Performance Status; LDH = lactate dehydrogenase; ULN = upper limit of normal; LLN = lower limit of normal

Parameters	Univariable Cox proportional hazards regression analysis		Multivariable Cox proportional hazards regression analysis		
					HR (95% CI)	P-value	HR (95% CI)	P-value	
	Smoking	1.45 (0.88-2.39)	0.14			
ECOG PS ≥2	4.41 (2.55-7.64)	<0.001	2.43 (1.18-5.00)	0.016	
Serum LDH >ULN	1.35 (0.74-2.47)	0.333			
Hemoglobin	0.40 (0.24-0.66)	<0.001	0.66 (0.35-1.25)	0.204	
Platelet count >ULN	3.69 (1.55-8.77)	0.003	1.59 (0.55-4.54)	0.391	
Absolute neutrophil count >ULN	1.03 (0.55-1.94)	0.92			
Absolute lymphocyte count	0.80 (0.49-1.34)	0.386			
≥2 visceral metastases	3.10 (1.64-5.88)	0.001	1.58 (0.70-3.55)	0.269	
Lung	0.43 (0.18-0.99)	0.049	0.41 (0.17-0.98)	0.044	
Bone	0.61 (0.34-1.08)	0.08			
Brain	0.39 (0.14-1.10)	0.076			
Liver metastasis	0.66 (0.36-1.21)	0.178			
Tumor type (clear vs. non-clear cell)	1.23 (0.73-2.07)	0.438			
Preoperative angioembolization	0.59 (0.32-1.13)	0.112			
Weight loss	0.36 (0.21-0.62)	<0.001	0.56 (0.29-1.9)	0.089	
Retroperitoneal lymphadenopathy	0.91 (0.55-1.5)	0.707			
Clinical evidence of renal	0.67 (0.40-1.10)	0.116			
Vein/caval thrombus					
Sarcomatoid differentiation	0.61 (0.32-1.15)	0.124			

## Discussion

The management of mRCC continues to evolve. Over the last two decades, there has been tremendous progress in the identification of prognostic factors which have a significant correlation with OS. The targeted therapy and risk stratification have significantly impacted the prediction of survival. There are variations in the demographic profiles of mRCC patients worldwide, which might have influenced the overall outcomes [1-10]. In this study, patients were aged 21-72 years, there was a male predominance, and all patients were symptomatic at presentation (Table 1). The classic triad of symptoms (flank pain, hematuria, and a palpable abdominal renal mass) reported by 9% of patients suggests locally advanced disease or metastatic disease [11]. The incidental detection of mRCC is reported in 10-21% of patients. The age range of 21-72 years with a median age of 50 years, male predominance (74.5%), and being symptomatic at presentation reflect poor health awareness and gender priority for treatment in our population (Table 1). Although the cause of mRCC in younger patients is unknown, it can be undetermined environmental, social, or geographic factors. Most patients (47.3%) were in the T3 stage, similar to previously reported studies [8,9,12]. The global incidence rate of mRCC varies. In India and other low-income countries such as Africa, the incidence rate of mRCC has been reported to be 2/100,000 and one female per 100,000 populations and 1/100000, respectively, much lower than Europe (16/100,000), the United States, and other western countries [13,14]. The Czech Republic has the highest incidence rates in the world (22/100,000 men and 9.9/100,000 women). With the exception of Israel, most African and Asian populations have the lowest rates [15]. Population-based research in the United States, Denmark, and the Netherlands demonstrate that lower socioeconomic level is associated with an increased prevalence of RCC. Poverty and education have also been shown to be determinants of the non-surgical management of African American patients with RCC [16-18].

Familial forms of RCC develop at an earlier age and are often multiple and bilateral. There is a 69% chance of developing RCC before age 60 in von Hippel-Lindau disease patients [19]. Acquired cystic kidney disease is also a significant risk factor. Cigarette smoking, hypertension, and obesity are strong modifiable risk factors, whereas diet and occupational factors are intermediate risk factors. Moreover, unusual medications are also a risk factor [20].

The mainstay treatment in our study was targeted therapy with a mean survival time of 21.52 months (standard error = 2.282 months, 95% CI = 17.040-25.985 months). Li et al. compared targeted therapy with no therapy in mRCC patients and observed that OS was 8.7 months (95% CI = 7.3-10.2 months) for targeted therapy versus 7.2 months in the no-therapy group [21]. Jürgens et al., in a comparative study between interferon therapy (IFNa) versus targeted therapy, showed significantly longer median survival in patients who received targeted therapy versus those who obtained INFa only (19.8 months (CI = 15.6-22.9) vs. 7.6 months (CI = 6.4-8.6); p < 0.001). Concerning adverse effects, patients on targeted therapy complained of nausea and vomiting (23%), loss of appetite (30%), and diarrhea (38%). However, 90% of these adverse effects were of grade 1 or 2, which is almost similar to a previously reported study [22].

Jürgens et al. observed that survival in clear cell carcinoma mRCC was 16.1 (12.9-19.5) months versus 7.0 (4.7-9.7) months in non-clear cell carcinoma (p = 0.002) [23]. In an exciting study reported by Schwab et al., in patients with non-clear cell histology (n = 18), including papillary and sarcomatoid variants, median OS was significantly inferior compared to clear cell histology (n = 99, 16.5 vs. 30.6 months; p < 0.05) [24]. The above results are similar to the results of our study, such as a survival time of 24.52 months (18.74-30.31) in clear cell versus 21.39 (13.323-29.457) months in papillary carcinoma. However, sarcomatoid differentiation significantly decreases survival time in both clear cell and papillary RCC. The mean OS in clear cell RCC was 24.52 months, whereas it reduced to 11.552 months when associated with sarcomatoid differentiation. In papillary RCC, the mean OS time was 21.390 months which was reduced to 7.370 months in those with sarcomatoid differentiation (Figure 2).

On analysis of survival based on the type of targeted therapy, the mean survival time was 23.10 months (14.445-31.762) for pazopanib-treated patients, 23.96 (15.481-32.432) months for sorafenib-treated patients, and 19.205 months (13.638-24.773) for sunitinib-treated patients (Figure 3). In the present study, the targeted therapy was given irrespective of the IMDC score. Thus, the OS might have been influenced as there were patients in favorable, intermediate, and poor-risk groups in all three types of targeted therapy groups. The above result is similar to the results reported by Santoni et al. on the outcome of patients treated with sorafenib, sunitinib, and pazopanib for late relapsing RCC with a median OS of 16.9 months in the sunitinib, 47.7 months in sorafenib, and 40.8 months in pazopanib groups, respectively [25]. When survival analysis was done based on the IMDC score, survival was maximum in the favorable-risk group and minimum in the poor-risk group. The mean survival time in the favorable (0 RF) group was 33 months (11.763-54.237), in the intermediate group (1-2 RF), it was 26.872 months (20.431-33.314), and in the poor group (>3 RF), it was 14.399 (8.851-19.947) months (p = 0.001). This was similar to the results reported by Jürgens et al. They observed that the OS was 28.4 (22.2-55.7) months in the favorable group, 13.2 (10.4-16.9) in the intermediate group, and 2.4 (1.1-8.5) months in the poor group (p < 0.001) [18]. In a study by Schwab et al., the median OS was 72.0 months for favorable-risk patients (n = 17, 95% CI = 1.20-2.77), 28.7 months for intermediate-risk patients (n = 79, 95% CI = 0.84-1.23), and 7.3 months for poor-risk patients (n = 28, 95% CI = 0.28-0.64; p < 0.01) [19]. Motzer et al. identified five predictive factors for mRCC survival analyses [26]. Low Karnofsky performance status (<80%), high serum lactate dehydrogenase (>1.5 times ULN), low hemoglobin (<LNL), high corrected serum calcium (>10 mg/dL), and absence of prior nephrectomy. These risk variables divided patients into three groups. Overall, 25% of patients with zero risk factors (favorable risk) lived for 20 months on average. Further, 53% of patients had one or two risk factors (intermediate risk), and median survival was 10 months. Poor-risk patients (22%) had a median survival time of four months. In a study of 120 mRCC patients with clear cell histology and ECOG PS 0 or 1, Choueiri et al. discovered five unfavorable prognostic factors. These included corrected serum calcium <8.5 or >10 mg/dL, absolute neutrophil count >4,500/dL, platelets >300,000/dL, RCC diagnosis to TT <2 years, and ECOG PS >0 [27]. The number of unfavorable prognostic variables determines three groupings. Patients with 0 or 1 unfavorable prognostic factor had a median PFS of 20.1 months compared to 13 months for those with two or more adverse prognostic factors. In a retrospective investigation of vascular endothelial growth factor targeted therapy patients, Heng et al. validated four MSKCC unfavorable prognostic variables as neutropenia and thrombocytosis [28]. Richey et al. used stepwise multivariable Cox proportional hazards regression analysis to predict worse survival in the overall group. LDH >ULN, corrected serum calcium >10.0 mmol/L, ECOG PS two or more, retroperitoneal lymph node metastasis (N2), platelet count >ULN, absolute lymphocyte count <LNL, two or more visceral/bone metastases, and a current smoker [5,29,30].

In the univariable Cox proportional hazards regression analysis, six factors remained independent for predicting an inferior survival in the entire cohort. These included ECOG PS ≥2, hemoglobin <LLN, platelet count >ULN, ≥2 visceral or bone metastases, lung metastasis, and weight loss. On multivariate Cox proportional hazard regression analysis, only lung metastasis and ECOG PS ≥2 were statistically significant (Table 2). The presence of these factors worsens the prognosis and decreases OS. To our knowledge, no previous prospective long-term study on mRCC has been reported from the Indian subcontinent.

## Conclusions

With the number of highly successful medicines available across various lines, developing a more customized approach to treatment selection will become increasingly critical. The IMDC and other clinical prognostic models have demonstrated a significant difference in OS. Furthermore, sarcomatoid differentiation was associated with a poor outcome. The study concluded that ECOG PS ≥2, hemoglobin <LLN, platelet count >ULN, ≥2 visceral or bone metastases, lung metastases, and weight loss are predictors of poor OS. These findings will aid in the development of future treatment approaches for the north Indian population.
